# Evaluation of a child food reward task and its association with maternal feeding practices

**DOI:** 10.1371/journal.pone.0254773

**Published:** 2021-07-21

**Authors:** Jia Ying Toh, Phaik Ling Quah, Chun Hong Wong, Wen Lun Yuan, Izzuddin M. Aris, Keri McCrickerd, Keith M. Godfrey, Yap-Seng Chong, Lynette P. Shek, Kok Hian Tan, Fabian Yap, Michael J. Meaney, Ciarán G. Forde, Yung Seng Lee, Birit F. P. Broekman, Mary F. F. Chong

**Affiliations:** 1 Singapore Institute for Clinical Sciences, Agency for Science, Technology, and Research, Singapore, Singapore; 2 Department of Food Science and Technology, National University of Singapore, Singapore, Singapore; 3 Department of Paediatrics, Yong Loo Lin School of Medicine, National University of Singapore, Singapore, Singapore; 4 Division of Chronic Disease Research Across the Lifecourse, Department of Population Medicine, Harvard Medical School and Harvard Pilgrim Health Care Institute, Boston, Massachusetts, United States of America; 5 Clinical Nutrition Research Centre, Singapore Institute for Clinical Sciences, Agency for Science, Technology and Research (A*STAR), National University Health System, Singapore, Singapore; 6 Medical Research Council Lifecourse Epidemiology Unit and National Institute for Health Research Southampton Biomedical Research Centre, University of Southampton and University Hospital, Southampton National Health Service Foundation Trust, Southampton, United Kingdom; 7 Department of Obstetrics & Gynaecology, Yong Loo Lin School of Medicine, National University of Singapore, Singapore, Singapore; 8 Divisions of Paediatric Allergy, Immunology, and Rheumatology, Khoo Teck Puat-National University Children’s Medical Institute, National University Hospital, National University Health System, Singapore, Singapore; 9 Maternal Fetal Medicine, KK Women’s and Children’s Hospital, Singapore, Singapore; 10 Duke-National University of Singapore Graduate Medical School, Singapore, Singapore; 11 Departments of Paediatrics, KK Women’s and Children’s Hospital, Singapore, Singapore; 12 Lee Kong Chian School of Medicine, Nanyang Technological University, Singapore, Singapore; 13 Integrative Neurosciences, Singapore Institute for Clinical Sciences, Agency for Science and Technology, Brenner Centre for Molecular Medicine, Singapore, Singapore; 14 Department of Psychiatry, McGill University, Montréal, QC, Canada; 15 Ludmer Centre for Neuroinformatics and Mental Health, Montréal, QC, Canada; 16 Department of Physiology, Yong Loo Lin School of Medicine, National University of Singapore, Singapore, Singapore; 17 Division of Paediatric Endocrinology, Khoo Teck Puat-National University Children’s Medical Institute, National University Hospital, National University Health System, Singapore, Singapore; 18 Department of Psychiatry, OLVG and AmsterdamUMC, VU University, Amsterdam, Netherlands; 19 Saw Swee Hock School of Public Health, National University of Singapore, Singapore, Singapore; Curtin University, AUSTRALIA

## Abstract

Food reward is defined as the momentary value of a food to the individual at the time of ingestion and is characterised by two psychological processes–“liking” and “wanting”. We aimed to validate an age-appropriate food reward task to quantify implicit wanting of children from the GUSTO cohort (n = 430). At age 5 years, child appetitive traits and maternal feeding practices were reported by mothers via questionnaires. At age 6, a write-for-food task based on the child’s preference for food or toy rewards was undertaken in laboratory conditions. Child BMI and skinfold measurements were taken at age 7. Convergent validity of the food reward task was assessed by associating with child appetitive traits, where enjoyment of food/food responsiveness (OR: 1.51; 95% CI: 1.06, 2.15) and emotional overeating (OR: 1.64; 95% CI: 1.09, 2.48) were positively associated with high food reward in children. Criterion validity was tested by associating with child BMI, however no significant relationships were observed. Multivariable logistic regression analysis with maternal feeding practices revealed that children whose mother tend to restrict unhealthy food (OR: 1.37; 95% CI: 1.03, 1.82) and girls whose mothers taught them about nutrition (OR: 2.09; 95% CI: 1.19, 3.67) were more likely to have high food reward. No further significant associations were observed between food reward, other appetitive traits and feeding practices. Despite the lack of association with child weight status, this study demonstrated the value of the write-for-food task to assess food reward in children and presented sex-specific associations with maternal feeding practices.

## Introduction

Food is a vital component of our lives and eating is one of several motivated behaviours that we carry out on a daily basis [1]. With the copious amounts of palatable food available in the current obesogenic environment, the motivation to eat can be driven by the reward value of the food item [2].

Defined as “the momentary value of a food to the individual at the time of ingestion”, food reward can be further characterised by two psychological processes–“liking” and “wanting” [3, 4]. Liking is the pleasurable sensation of eating a food and wanting is the compulsion to eat triggered by a food cue [5]. These processes influence food consumption to varying degrees, with liking accounting for a small percentage of the variance in intake, while wanting appears more dominant when in the presence of palatable food [6].

While a variety of methodologies has been used to measure food reward, differences lie in their degrees of explicitness. Measures of explicit wanting typically depends on subjective self-reports [7], where participants rate their wanting for a food after tasting or seeing its picture [8, 9]. On the other hand, implicit wanting depends on participants’ willingness to work for rewards, where the amount of work done corresponds to the value of the reward [7] e.g. forced choice reaction time paradigm of the Leeds Food Preference Questionnaire (LFPQ) [10–14] and progressive ratio computer task [15–18]. It is recommended that the measures of implicit wanting be applied more as they are better able to characterise implicit motivational process [6]. However, compared to adults, studies measuring the ‘wanting’ component of food reward in children are limited. Current studies mostly adopt the progressive-ratio computer task [19], which may not be straightforward to develop and easy to comprehend in young children [17].

In this study, we developed an age-appropriate food reward task to understand the implicit decision-making processes that children take when given a choice to exert efforts to obtain their desired food object or non-food object. Children’s food reward behaviour, defined as willingness to work for food rewards, will be measured. To demonstrate validity of this food reward task, we assessed its convergent validity by correlating it with food-approach traits that have been commonly captured in other studies such as the parent-reported child eating behaviour questionnaire [20]. An example is food responsiveness, which is the tendency to eat in response to environmental food cues. In children aged 2.5–9 years, this has been associated with greater sensitivity to the reward value of palatable foods, as measured by the Behavioural Inhibition System/Behavioural Approach System (BIS/BAS) scales [21]. We hypothesized that greater willingness to work for food rewards in children will be associated with food-approach traits in children as reported by parents.

We also assessed the task’s criterion validity by relating it to weight status and adiposity measures, where we hypothesized that children who tend to work for food rewards are more likely to be overweight/obese. Greater willingness to work for food reward has been associated with higher body weight, mediated by increased energy intake [22, 23]. For example, individuals who are overweight or have obesity display higher motivation to work for food relative to non-food alternatives, such as stickers, money or time to read or play video games, compared to their leaner peers. This appears to be consistent throughout different age groups including infancy [24], childhood [25–27], adolescence [16] and adulthood [15, 18, 28, 29]. Prospective studies in children, adolescents and adults have shown that food reward behaviour predicted weight gain at 1 to 2 years follow up, suggesting food reward behaviour as a risk factor for obesity [16, 27, 29].

There is evidence to suggest that parental feeding practices may influence food reward behaviour in children. Children aged 2–7 years of age who experienced parental restriction of sweet and savoury snack foods were observed to consume more of these restricted snacks when compared to children who had less parental restrictions [30–32]. We hypothesized that parental feeding restrictions could paradoxically increase food reward behaviour in their offspring. Thus, lastly, we also examined demographic and other parental feeding practices that may influence food reward behaviour in children.

## Materials and methods

### Study design and participants

Data for the present analysis was obtained from the Growing Up in Singapore Towards healthy Outcomes (GUSTO) study, a prospective mother-child dyad cohort study aimed at investigating early developmental pathways during pregnancy, infancy and childhood and their effect on metabolic disease outcomes. GUSTO was established in June 2009, where women in the first trimester of pregnancy aged 18 to 50 years old were recruited for the study from two major hospitals in Singapore, National University Hospital (NUH) and KK Women’s and Children’s Hospital (KKH). Women suffering from type I diabetes mellitus, cancer or receiving psychotropic drugs were excluded from the study. Both mother and child attended home visits every 3 months from birth till 18 months postnatal and clinic visits annually from 2 years of age. Further details of the study have been previously described [33]. Written informed consent were obtained from mothers during clinic visits at ages 5 and 6-years. This study was approved by the National Healthcare Group Domain Specific Review Board and the Sing Health Centralized Institutional Review Board.

### Maternal reports of children’s appetitive traits

The Child Eating Behaviour Questionnaire (CEBQ) is a parent reported questionnaire constructed by Wardle et al. to measure children’s appetitive traits [20]. The questionnaire consists of 35 items measuring 4 food-approach appetitive traits (food responsiveness, enjoyment of food, emotional overeating, and desire to drink) and 4 food-avoidant appetitive traits (slowness in eating, satiety responsiveness, food fussiness, and emotional undereating). The CEBQ has previously been validated in the GUSTO cohort and showed that a revised 6-factor structure was more appropriate for local children 5 to 6 years of age. Food fussiness, desire to drink, emotional undereating and emotional overeating factors were mostly retained, while food responsiveness merged with enjoyment of food (enjoyment of food / food responsiveness) and satiety responsiveness merged with slowness in eating (slowness in eating / satiety responsiveness). Cronbach’s α estimates of the 6 domains ranged from 0.70–0.85, suggesting good internal consistency [34]. In this study, maternal reported CEBQ data was collected when the children were 5 years old. Mean scores were calculated from each of the 6 domains, where a higher mean score indicated a greater adherence to the appetitive trait.

### Self-reported maternal feeding practices

The Comprehensive Feeding Practices Questionnaire (CFPQ), a self-reported validated questionnaire [35], was used to examine maternal feeding practices at the 5-year clinic visit. It consists of 49 items measuring 6 domains of positive feeding practices (encourage balance/variety, teaching nutrition, healthy environment, involvement, modelling and monitoring) and 6 domains of negative feeding practices (restriction for health, restriction for weight, child control, emotion regulation, food as a reward and pressure to eat). Cronbach’s α estimates of all the domains ranged from 0.56–0.86, suggesting moderate to high internal consistency [36]. Mean scores were calculated from each domain, where a higher mean score indicated a greater adherence to the feeding practice domain.

### Child anthropometric measurements

Child anthropometric measurements were recorded at 7 years of age by trained clinic staff. Weight was measured using calibrated scales (SECA 813), while standing height was measured using a stadiometer (SECA 213). Based on WHO Child Growth Standards 2006, age and sex-adjusted BMI z-scores were derived using the WHO AnthroPlus software (Version 3.2.2) [37]. Children were further categorised as underweight (< -2SD); normal weight (-2SD to +1SD); overweight or obese (> +1SD). Skinfold measurements were taken from the right side of the body, using skinfold callipers (Holtain Ltd). All anthropometric measurements were taken in duplicates, except skinfold measurements which were taken in triplicates, and averaged.

### Food reward—Write-for-food task

In this task, the propensity to write for food as compared to a non-food alternative was measured by how willing 6-year-old children were to hand-write rows of symbols to obtain either a food or non-food (toy) reward or both. This task operationalises implicit wanting as the amount of work the child is willing to put in to obtain the reward. The task was conducted at the end of a neurocognitive study clinic visit, approximately 1-hour after an *ad libitum* meal to ensure that the motivation was not driven by hunger.

The food items were shortlisted from a validated food frequency questionnaire interviewer-administered to caregivers when children were 5 years of age [38], while toys of affordable value were shortlisted based on gifts provided for and shown to be popular in previous experimental tasks for this particular age group of children. Using the five most commonly consumed snacks and popular toys reported, a pilot study (n = 46) was conducted with children between 5 to 10 years of age to further narrow down the rewards to three items each. The children in the pilot study were presented with the shortlisted foods and toys and were asked to rank their top three selections respectively. The final rewards used for the main study were cookies, potato chips and sweets for food items and scratch paper, crayons and plasticine for toys.

At the beginning of the task, each child was presented with the food and toy items and asked to select his/her most preferred food and toy. Verbal and pictorial instructions to perform the task were provided next. These were counterbalanced among the children (e.g. alternate between talking about food/toy first). The child was given the option to write for either a food item or a toy, or both items by doing some writing. The child was given 10 minutes to write symbols (“$” representing food; “%” representing toy) on two separate worksheets. Each worksheet contained 15 rows, with 10 empty boxes in each row. At the end of 10 minutes, the child was given a portion of his/her chosen food and/or toy e.g. 1 piece of cookie or 1 stick of crayon for each completed row (i.e. 10 filled boxes). If the child completed all 15 rows, he/she was rewarded with the food item and/or toy in its entirety e.g. 1 box of cookies or 1 set of crayons. Pictorial instructions of the task can be found in S1 Fig of S1 File. The experimenter checked whether the child understood the instructions and repeated the steps when necessary. If the child was unable to comprehend the task, he/she was allowed to write for 5 minutes and the task was then terminated. The child still received his/her chosen item but data were excluded from this study. If the child showed an understanding of the task, the experimenter proceeded to practice writing with the child by allowing him/her to trace the “$” and “%” symbols in the first 3 boxes of each piece of worksheet. At the end of the practice session, the experimenter left the room and the child was given 10 minutes to write. Each child could choose to write for the full 10 minutes or alert the experimenter if he/she decided to stop writing. The experimenter collected the worksheets and checked the child’s understanding of the task again by asking a series of questions.

**Fig 1:**
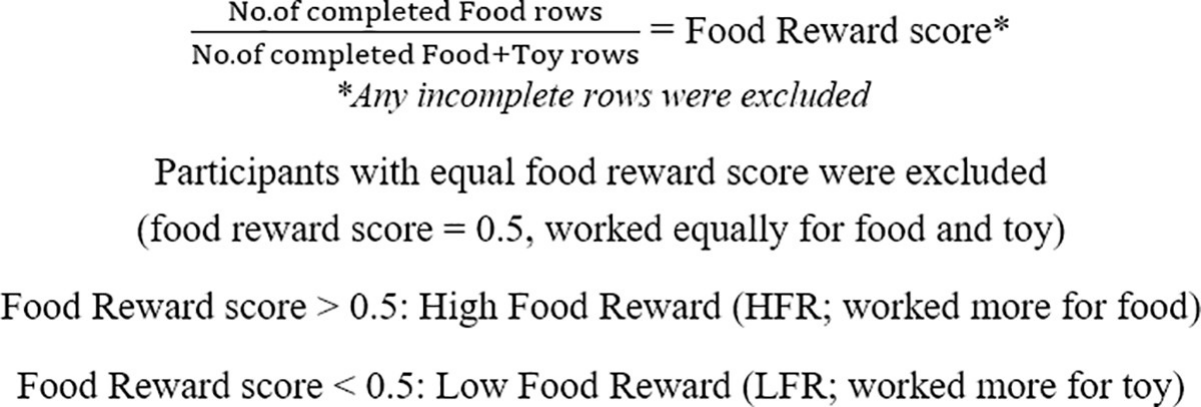
Calculation and categorisation of food reward score.

The number of completed rows written for the chosen item(s) was later recorded down by the researcher and any incomplete rows excluded. Food reward was calculated by dividing the number of completed rows written for food by the total number of completed rows written for both food and/or toy. Using the total number of rows written for both food and toy as reference (i.e. denominator), would provide a reliable measure of food reward as it accounted for the child’s choice to write for both food and toy [39]. Children who wrote more rows for food than non-food would have scored greater than 0.5 and were identified to have a higher response for the write-for-food task i.e. high food reward (HFR). Those who wrote fewer rows for food than for non-food would have scored less than 0.5 and were deemed to have a lower response for the write-for-food task i.e. low food reward (LFR). Those with scores equal to 0.5 were classified as “wrote equally for food and toy” (Fig 1).

### Covariates

Age, ethnicity, household income, education and marital status of mothers were collected during recruitment and at 26–28 weeks of gestation. Maternal weight (SECA 803) and height (SECA 213) were measured during the Year 6 clinic visit and used to calculate body mass index (BMI) (kg/m^2^). Child gestational age was determined from an ultrasound scan in the first trimester, while information on sex and birth order was obtained from delivery records. Child BMI at 5 years of age was calculated from height and weight measured during the Year 5 clinic visit and were subsequently converted to z-scores as well.

### Statistical analyses

Of the 1247 mother-child pairs recruited, 556 children participated in the write-for-food task at age 6 years. Of the 556 children, 72 of them wrote equally for food and toy. To examine food reward, we chose to focus on comparing children who wrote more for food (high food reward, HFR; n = 245) against those who wrote more for toy (low food reward, LFR; n = 185) for the main analyses. After excluding twins (n = 7) and children who did not adhere to protocol (n = 47), the final study sampled consisted of 430 singleton children.

Differences in characteristics between HFR and LFR groups were analysed using independent samples t-tests for maternal age (years), maternal BMI (kg/m^2^), child gestational age (weeks) and child BMI z-score at 5 years, while maternal BMI groups, ethnicity, household income, education, marital status, parity, child BMI z-score groups and sex were analysed using chi-square tests. Potential differences in characteristics between included and the excluded participants were also analysed. Convergent validity was assessed by associating HFR and LFR with CEBQ subscale scores using multivariable logistic regression, while criterion validity was assessed by associating HFR and LFR with child anthropometry outcomes at 7 years of age using multivariable linear regression and multinomial logistic regression. Multivariable logistic regression was also used to examine the associations with CFPQ subscale scores (maternal feeding practices) with children of high food reward compared to those with low food reward, and applied the following models–Model 1: unadjusted; Model 2: Model 1 and additional adjustment for ethnicity, household income, maternal age and child BMI z-scores at 5 years; and stratified models.

Covariates were identified based on significant characteristics differences, contributing to ≥ 5% change in estimates at univariate level [40] and literature findings [25, 31, 39, 41–43]. Stratification was determined through significant interaction observed between CEBQ, CFPQ subscales and sex (CEBQ subscales × Sex; CFPQ subscales × Sex) in model 2. Missing covariate data [Maternal BMI (n = 61); Household Income (n = 25); Education (n = 6); Marital Status (n = 11); Child BMI z-scores at 5 years (n = 17)] were estimated by fully conditional specification multiple imputation [44]. Fifty chained equation imputations were generated and the pooled analyses were presented. Sensitivity analysis was conducted using complete data to identify any potential effects of multiple imputation. Sub-group analysis for children who wrote equally for food and toy (n = 72) was also conducted using multinomial logistic regression with LFH as reference group. All statistical analyses were performed using SPSS version 23.0 (IBM Corp, New York, NY, USA). Two-sided p-values <0.05 were considered statistically significant.

## Results

### Study sample characteristics

Of the 430 children, 57% (n = 245) chose to write more for a food reward than a toy reward. Children living in families with the highest household income tended to have HFR, whereas children of middle income families tended to have LFR. Children of Chinese ethnicity tended to have HFR than LFR, but no differences in food reward were observed between children of Malay and Indian ethnicity. There were no significant differences in maternal characteristics (maternal age, maternal BMI at 6-year clinic visit, education, marital status and parity) between HFR and LFR children. There were also no significant differences observed for child sex, gestational age and BMI z-scores at 5 years between HFR and LFR (Table 1).

**Table 1:** Maternal and child characteristics.

	High Food Reward (n = 245)		Low Food Reward (n = 185)		*p-value*
	Mean / n	SD / %	Mean / n	SD / %	
*Maternal Characteristics*					
**Age (years)**	31.7	5.0	31.2	5.0	0.28
**BMI at year 6 clinic visit (kg/m**^**2**^**)**	25.0	6.3	25.7	6.6	0.27
**BMI groups at year 6 clinic visit**					0.17
Underweight (< 18.5)	16	6.5	10	5.4	
Normal weight (18.5–24.9)	127	51.8	88	47.6	
Overweight (25.0–29.9)	58	23.7	49	26.5	
Obese (≥ 30)	44	18.0	38	20.5	
**Ethnicity**					0.06
Chinese	152	62.0^a^	94	50.8^b^	
Malay	57	23.3^a^	54	29.2^a^	
Indian	36	14.7^a^	37	20.0^a^	
**Household Income**					0.02
< $2000	31	12.7^a^	24	13.0^a^	
$2000 –$5999	123	50.2^a^	115	62.2^b^	
≥ $6000	91	37.1^a^	46	24.9^b^	
**Education**					0.72
Secondary and below	68	27.8	49	26.5	
Post-Secondary	88	35.9	69	37.3	
University and above	89	36.3	67	36.2	
**Marital Status**					0.42
Married	234	95.5	180	97.3	
Single / Divorced	11	4.5	5	2.7	
**Parity**					0.31
Primiparous	106	43.3	90	48.6	
Multiparous	139	56.7	95	51.4	
*Child Characteristics*					
**Gestational age (weeks)**	38.7	1.8	38.8	1.3	0.46
**BMI z-score at 5 years old**	0.1	1.3	0.1	1.4	0.82
**BMI groups at 5 years old**					0.46
Underweight (< -2SD)	6	2.4	3	1.6	
Normal weight (-2 SD to 1SD)	196	80.0	150	81.1	
Overweight / Obese (> +1SD)	43	17.6	32	17.3	
**Sex**					0.11
Male	138	56.3	89	48.1	
Female	107	43.7	96	51.9	

*p-values* were obtained from independent samples t-test for continuous variables and chi-squared tests for categorical variables

^a,b^ Values in the same row not sharing the same subscript are significantly different at p < 0.05 based on Bonferroni correction

Column (%) adds up to 100%

Imputed variables: Maternal BMI (n = 61); Household Income (n = 25); Education (n = 6); Marital Status (n = 11); Child BMI z-scores (n = 17)

Sub-group analysis of children who wrote equally for food and toy tended to be Malay, have mothers with secondary or lower education and less likely to be living in families with high household income (S1 Table in S1 File).

### Convergent validity

Children with higher enjoyment of food/food responsiveness on the CEBQ were 1.53 times more likely to have HFR during the lab task (95% CI: 1.10, 2.11). Similar associations were observed for children with higher emotional overeating (Odds Ratio (OR): 1.63; 95% CI: 1.09, 2.43). These relationships remained significant after adjusting for confounders (Table 2). No associations were observed for the remaining CEBQ subscales and no significant interactions were observed between all the CEBQ subscales and sex (p-interaction > 0.05).

**Table 2:** Associations between child appetitive traits at 5 years old and food reward at 6 years old (n = 326).

	Model 1	Model 2
	OR (95% CI)	OR (95% CI)
*Food-Approach Traits*		
Enjoyment of food / Food responsiveness	1.53 (1.10, 2.11)*	1.51 (1.06, 2.15)*
Emotional overeating	1.63 (1.09, 2.43)*	1.64 (1.09, 2.48)*
Desire to drink	1.20 (0.92, 1.55)	1.22 (0.93, 1.59)
*Food-Avoidant Traits*		
Slowness in eating / Satiety responsiveness	0.76 (0.55, 1.04)	0.75 (0.53, 1.05)
Food fussiness	0.87 (0.65, 1.16)	0.86 (0.64, 1.16)
Emotional undereating	1.06 (0.77, 1.45)	1.04 (0.74, 1.44)

Model 1: Unadjusted

Model 2: Adjusted for ethnicity, household income, maternal age and child BMI z-scores at 5 years old

*Significant at p < 0.05

The results were generally similar when adjusted logistic regression models were restricted to participants with no missing covariate data (CEBQ: n = 309). The associations of enjoyment of food/food responsiveness (OR: 1.47; 95% CI: 1.02, 2.11) and emotional overeating subscale (OR: 1.61; 95% CI: 1.06, 2.44) with food reward were slightly attenuated (S2 Table in S1 File). Sub-group analysis of child appetitive traits with children who wrote equally largely yield no significant associations (S3 Table in S1 File).

### Criterion validity

In adjusted models, there were no statistically significant associations observed between food reward at 6 years of age and child skinfolds at 7 years of age. No significant associations were also observed with child BMI z-scores and weight status at 7 years of age (Tables 3 and 4). Associations observed in sensitivity and sub-groups analyses were also not statistically different (S4 –S7 Tables in S1 File).

**Table 3:** Associations between food reward at 6 years old and child adiposity at 7 years old (n = 398).

	Total Skinfolds (mm)	Subscapular (mm)	Triceps (mm)	Biceps (mm)	Suprailiac (mm)	BMI (z-scores)
	β (95% CI)	β (95% CI)	β (95% CI)	β (95% CI)	β (95% CI)	β (95% CI)
High Food Reward^+^	1.75 (-1.18, 4.69)	0.63 (-0.18, 1.44)	0.51 (-0.33, 1.35)	0.16 (-0.38, 0.69)	0.45 (-0.46, 1.37)	0.19 (-0.07, 0.46)

Adjusted for ethnicity, household income, maternal BMI, maternal age, parity, gestational age

^+^Reference: Children with Low Food Reward data 7 years old (n = 170)

**Table 4:** Associations between food reward at 6 years old and child adiposity at 7 years old (n = 398).

	Underweight (n = 12)^^^	Overweight / Obese (n = 93)^^^
	OR (95% CI)	OR (95% CI)
High Food Reward^+^	1.61 (0.46, 5.65)	1.44 (0.86, 2.41)

Adjusted for ethnicity, household income, maternal BMI, maternal age, parity, gestational age

^+^Reference: Children with Low Food Reward data 7 years old (n = 170)

^^^Reference: Normal weight status at 7 years old (n = 293)

### Association of maternal feeding practices and food reward

In both crude and adjusted models, mothers who tended to restrict intake of unhealthy foods had children who were more likely to have HFR (Model 2 OR: 1.37; 95% CI: 1.03, 1.82). No associations were observed between the other maternal feeding practices (CFPQ subscales) and food reward. However, significant interactions between “Encourage balance/variety” (p-interaction < 0.01), and “Teaching nutrition” (p-interaction = 0.01) subscales and sex in relation to food reward were found. Therefore, sex-specific associations of these subscales and food reward were further explored.

In stratified model 2, mothers who encouraged balance/variety had boys who were less likely to have HFR (OR: 0.50; 95% CI: 0.27, 0.92). No significant association was seen in girls. Mothers that tended to teach nutrition had girls who were 2.09 times more likely to have HFR (95% CI: 1.19, 3.67). No other significant association was seen in boys (Table 5).

**Table 5:** Associations between maternal feeding practices at 5 years old and food reward at 6 years old (n = 307).

	Model 1	Model 2	*p-interaction*	Model 2: Male (n = 165)	Model 2: Female (n = 142)
	OR (95% CI)	OR (95% CI)		OR (95% CI)	OR (95% CI)
*Positive Feeding Practices*					
Encourage balance/variety	1.01 (0.68, 1.51)	0.92 (0.61, 1.40)	*< 0*.*01*	0.50 (0.27, 0.92)*	1.71 (0.89, 3.26)
Teaching nutrition	1.27 (0.93, 1.73)	1.23 (0.89, 1.71)	*0*.*01*	0.86 (0.55, 1.33)	2.09 (1.19, 3.67)*
Healthy environment	0.93 (0.69, 1.25)	0.85 (0.62, 1.18)	*0*.*22*		
Involvement	0.99 (0.77, 1.27)	1.02 (0.78, 1.33)	*0*.*99*		
Modelling	1.04 (0.82, 1.31)	0.99 (0.78, 1.27)	*0*.*13*		
Monitoring	0.97 (0.77, 1.23)	0.98 (0.77, 1.24)	*0*.*25*		
*Negative Feeding Practices*					
Restriction for health	1.41 (1.07, 1.86)*	1.37 (1.03, 1.82)*	*0*.*77*		
Restriction for weight	1.05 (0.82, 1.36)	1.04 (0.79, 1.38)	*0*.*22*		
Child control	1.21 (0.84, 1.75)	1.23 (0.84, 1.82)	*0*.*68*		
Emotion regulation	0.90 (0.67, 1.23)	0.93 (0.68, 1.27)	*0*.*29*		
Food as a reward	1.05 (0.83, 1.32)	1.07 (0.84, 1.36)	*0*.*21*		
Pressure to eat	1.20 (0.89, 1.61)	1.15 (0.84, 1.58)	*0*.*18*		

Model 1: Unadjusted

Model 2 and stratified models: Adjusted for ethnicity, household income, maternal age and child BMI z-scores at 5 years old

*Significant at p < 0.05

In models with no missing covariate data (CFPQ: n = 292), association with restriction for health remained (OR: 1.36; 95% CI: 1.02, 1.83). When stratified by sex, the positive association of teaching nutrition strengthened in girls (OR: 2.38; 95% CI: 1.32, 4.28), while the association of encouraging balance/variety (OR: 0.52; 95% CI: 0.28, 0.96) remained similar in boys. (S8 Table in S1 File). Stratified sub-group analysis showed fewer significant associations between maternal feeding practices and children who wrote equally (S9 Table in S1 File).

## Discussion

The present study provided some evidence of convergent validity for the write-for-food task, where children who were reported to enjoy food/be more food responsive and to overeat when emotional were more likely to have higher food reward. However, there was a lack of evidence of any association with child weight status, hence criterion validity could not be determined. Some significant associations with maternal feeding practices were observed–children whose mother tended to restrict unhealthy foods were more likely to have higher food reward. When stratified by sex, boys whose mothers encouraged balance and variety in their diet were less likely to have high food reward, but this was not observed in girls. However, girls whose mothers provided education on nutrition were more likely to have high food reward, but such association was not observed in boys.

Amongst the various child appetitive traits examined, enjoyment of food/food responsiveness and emotional overeating emerged to be the only two appetitive traits that were positively associated with food reward. Enjoyment of food/Food responsiveness is characterised by a child’s overall appetite for food and their inclination to eat based on the influence of external food prompts, while emotional overeating describes a child who increases his/her food consumption due to the influence of negative emotions [20, 45, 46]. When these two appetitive traits are strong, food plays a reinforcing role, motivating the child to eat rather than engaging in other non-food activities. In this way, food has a stronger rewarding value among these children. Whether these three eating behaviour dimensions are conceptually independent or overlapping in children remains unclear [47] and the current study is the first to demonstrate that these dimensions are closely related in children, providing evidence that the write-for-food task could potentially be used to assess food reward in children.

No associations were observed between food reward and desire to drink. This was not unexpected as the reward items offered as part of the study were snack items rather than beverages. In addition, no associations were observed between food reward and the four food-avoidant traits. While reverse associations would have been expected, the lower odd ratios for these traits do suggest that those with food-avoidant traits tended to have lower likelihood of having high food reward, although findings were not statistically significant.

We did not find associations between food reward and being overweight/obese in our study, as reflected by the null associations with BMI. In our study sample, the majority of children were of normal weight (73%). In contrast to existing literature focusing mostly on overweight, obesity and higher food reward [19], our study suggests that normal weight range children can have high food reward, similar to findings observed by Kral et al, who measured food reward using the relative reinforcing value of food task [48]. There is also evidence suggesting an ‘inverted-U’ relationship between reward sensitivity and BMI in adults [49]. Our findings also support a recent study conclusion that children’s brain responses to food and monetary rewards were independent of their weight status [50]. Whether this places normal weight children that are higher in food reward at a greater risk of weight gain and obesity in later childhood remains to be further investigated.

Higher food reward could also be due to the influence of mother’s feeding practices on child eating behaviour. Several studies observed that maternal restrictive feeding practices were associated with increased consumption of snack foods in children [30–32], which is in line with our findings that restriction of unhealthy food is associated with high food reward in children. However, we did not find any association between restriction of food for weight control and high food reward in children. This is not unexpected as majority of the children in our study sample is of normal weight range, hence it is possible that mothers are less concerned about their child’s weight status. While some studies have reported that children who were frequently given food as a reward were found to be more food responsive and more likely to emotionally overeat [51–53], our study did not observe an association between mothers providing food as rewards and a greater sense of value of food in their offspring. It is possible that mothers responded to the questions on feeding practices based on their own perception but may not have done so to a significant extent in practice, thus resulting in the null associations [54]. These findings are also in line with findings from our previous study conducted in the GUSTO cohort, where maternal reported food reward feeding practices were not found to be associated with child’s intakes of discretionary foods such as sweet snacks [54].

Instead, we found that the practice of teaching children about nutrition was associated with high food reward among females, but not among males. Sex differences in observed maternal feeding practices have also been previously examined in GUSTO children at 4.5 years of age, where mothers were reported to display more controlling feeding practices in response to girls who ate faster, took fewer chews and had larger bite size. This was however, not observed in boys [55]. It appears that girls are generally more prone to experience parental weight control and restrictive feeding practices, perhaps due to greater cultural and societal expectation for girls to control their weight, compared to boys [56–58]. Yet, studies have observed that with greater parental restriction, girls are more likely to eat in the absence of hunger [57, 59], and overconsume snacks when the opportunity is given [60], resulting in eventual weight gain [59].

It is also possible that while mothers in our study reported imparting nutrition knowledge, they could unconsciously and/or unintentionally have restricted access to unhealthy foods, as observed from the weak but significant correlation between these domains (r = 0.15) (S10 Table in S1 File). This may result in children, especially girls, exhibiting a greater tendency to work for such foods when unrestricted. Such inconsistencies in reported and observed maternal behaviour were highlighted by Scaglioni et al., suggesting that mothers may conscientiously verbalise or demonstrate healthy eating habits for their child, but unconsciously model unhealthy snacking habits [41].

This study has its strengths and limitations. The value of the food reward task is that it is an objective measure that is independently completed by the child. This is unlike the parent-reported appetitive traits, where the information may be subjective and may not fully reflect a child’s own motivation towards food as a reward. Thus, the food reward task is more likely to reflect a child’s own motivation towards food as a reward compared to a non-food. Another strength to highlight would be the sample size of our study. With a study sample of 430 children, it is comparatively larger than most of the existing studies that have employed food reward tasks (sample size ranges from n = 30 to n = 273) [16–18, 22, 24, 31, 48]. In terms of the limitations of this study, it is recognised that the measurement of food reward is complex as its components–wanting and liking, are unobservable and cannot be measured directly [61]. As such, there is currently no consensus on the definition of the components of food reward [6]. Temporality could not be established due to the short follow-up between administering the questionnaires and conducting the write-for-food task. In addition, reverse causality could be present as mothers could have responded to their more food responsive children by proactively teaching them about nutrition and encouraging balance/variety. The Cronbach’s α estimates of some parental feeding practices domains were below the acceptable value of 0.7 (Teaching nutrition Cronbach’s α = 0.56). However, estimates close to 0.6 are acceptable if the domain consists of a few items (e.g. Teaching nutrition contains 3 items only) [36, 62]. Social and recall bias could also be present in the self-administered questionnaires, as data was collected retrospectively and there is a possibility that parents would choose to over-report ratings of healthy feeding practices [63], prompting the need for further research to objectively assess feeding practices that are socially deemed desirable or undesirable [63, 64]. Furthermore, in Singapore, parents are not the sole caregivers of the children. It is reported that about 20% of Singaporean families rely on grandparents as the main caregiver and about 33% of grandparents care for their grandchildren regularly [65]. In our study, 12% of primary caregivers were grandparents and 34% shared the responsibility of caring for the child with another caregiver. Besides potential differences in feeding practices between parents and grandparents, there could be under-reporting of child appetitive traits. Obtaining information from the grandparents could provide a more accurate understanding of the child’s appetitive trait and the feeding practices applied in a Singaporean household. In view of the subjectivity of parent-reported data, further validation against other objective measures of food reward such as the food reinforcement task [16], Leeds Food Preference Questionnaire [10] or fMRI scans of brain responses towards various reward types [50] could help establish more robust conclusions.

## Conclusions

In conclusion, the write-for-food task demonstrated some potential for assessing food reward in children. However, the lack of associations with child adiposity and certain maternal feeding practices meant that criterion validity of this food reward task and concurrence with some existing literature have yet to be established. As such, further investigation of the validity of this food reward task is warranted and preferably against other objective and established measures of food reward tasks, prior to its further application in studies examining food reward in children.

## Supporting information

S1 File Supporting information (S1 Fig, S1 –S10 Tables). (PDF)
