# Supplementary material for: Evaluation of a child food reward task and its association with maternal feeding practices
Source: PLoS One. 2021 Jul 21;16(7):e0254773. doi: 10.1371/journal.pone.0254773 (PMC8294516; doi:10.1371/journal.pone.0254773)
Supplement: S1 File — (PDF) [file pone.0254773.s001.pdf]

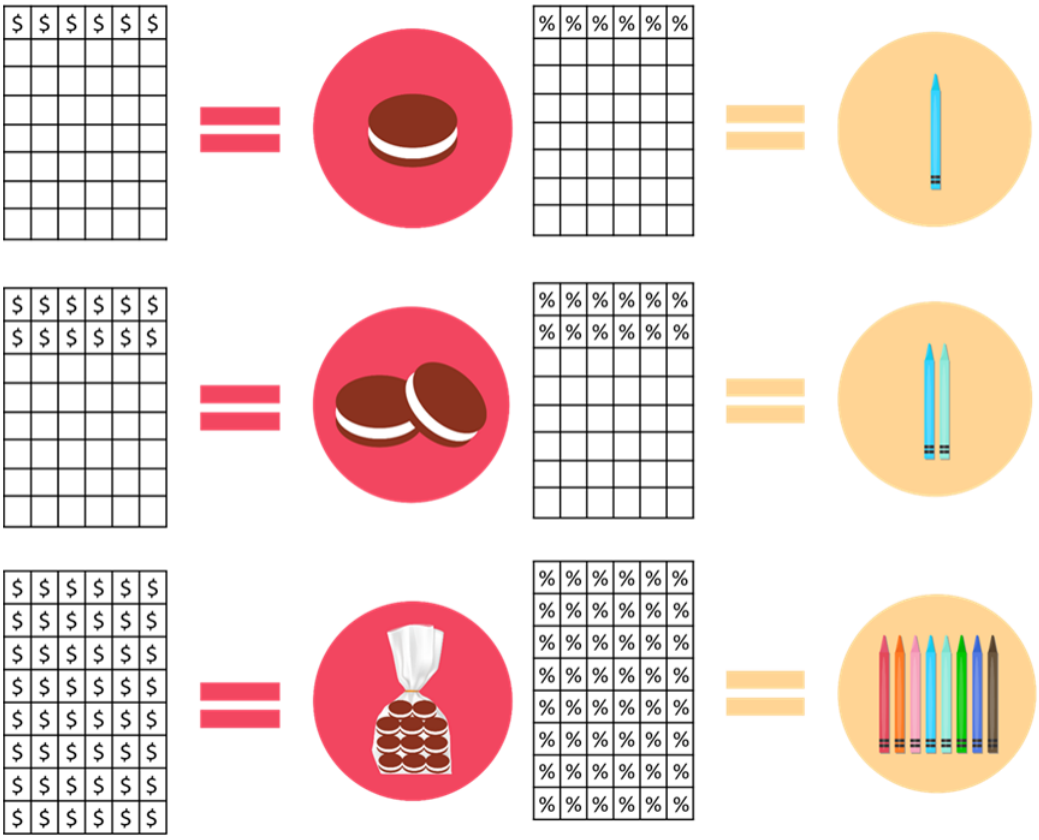

S1 Fig: Pictorial Instructions of Food Reward Task

S1 Table: Maternal and child characteristics (n = 502) (Unimputed)

	Wrote more for food or toy (n = 430)		Wrote equally (n = 72)		*p-value*
	Mean / n	SD / %	Mean / n	SD / %	
Maternal Characteristics					
**Age (years)**	31.5	5.0	32.1	5.8	*0.40*
**BMI at year 6 clinic visit (kg/m^2)+**	25.2	5.5	24.5	4.2	*0.25*
**BMI groups at year 6 clinic visit+**					*0.46*
Underweight (< 18.5)	18	4.9	2	3.4	
Normal weight (18.5 – 24.9)	192	52.0	33	55.9	
Overweight (25.0 – 29.9)	93	25.2	18	30.5	
Obese (≥ 30)	66	17.9	6	10.2	
**Ethnicity**					*0.03*
Chinese	246	57.2[a]	35	48.6[a]	
Malay	111	25.8[a]	29	40.3[b]	
Indian	73	17.0[a]	8	11.1[a]	
**Household Income+**					*0.07*
< $2000	51	12.6[a]	13	18.8[a]	
$2000 – $5999	226	55.8[a]	43	62.3[a]	
≥ $6000	128	31.6[a]	13	18.8[b]	
**Education+**					*0.01*
Secondary and below	115	27.1[a]	32	44.4[b]	
Post-Secondary	154	36.3[a]	22	30.6[a]	
University and above	155	36.6[a]	18	25.0[a]	
**Marital Status+**					*0.72*
Married	408	97.4	68	95.8	
Single / Divorced	11	2.6	3	4.2	
**Parity**					*0.96*
Primiparous	196	45.6	32	44.4	
Multiparous	234	54.4	40	55.6	
Child Characteristics					
**Gestational age (weeks)**	38.7	1.6	39.1	1.1	*0.05*
**BMI z-score at 5 years old+**	0.1	1.3	0.0	1.0	*0.40*
**BMI groups at 5 years old+**					
Underweight (< -2SD)	332	80.4	59	85.5	*0.55*
Normal weight (-2 SD to 1SD)	6	1.5	1	1.4	
Overweight / Obese (> +1SD)	75	18.2	9	13.0	
**Gender**					*0.93*
Male	227	52.8	39	54.2	
Female	203	47.2	33	45.8	

p-values were obtained from independent sample t-test for continuous variables and chi-squared tests for categorical variables

[a,b] Values in the same row not sharing the same subscript are significantly different at $p < 0.05$ based on Bonferroni correction

Column (%) adds up to 100%

+Missing data: Maternal BMI (n = 74); Household Income (n = 28); Education (n = 6); Marital Status (n = 12); Child BMI z-scores (n = 20)

S2 Table: Associations between child appetitive traits at 5 years old and food reward at 6 years old (n = 309) (Unimputed)

	Model 2
	OR (95% CI)
Food-Approach Traits	
Enjoyment of food / Food responsiveness	1.47 (1.02, 2.11)*
Emotional overeating	1.61 (1.06, 2.44)*
Desire to drink	1.16 (0.88, 1.53)
Food-Avoidant Traits	
Slowness in eating / Satiety responsiveness	0.78 (0.55, 1.12)
Food fussiness	0.89 (0.66, 1.20)
Emotional undereating	1.08 (0.77, 1.52)

Adjusted for ethnicity, household income, maternal age and child BMI z-scores at 5 years old

*Significant at $p < 0.05$

S3 Table: Associations between child appetitive traits at 5 years old and food reward at 6 years old (n = 386) (High Food Reward, Low Food Reward and wrote equally)

	High Food Reward (n = 182)+	Wrote Equally (n = 60)+
	OR (95% CI)	OR (95% CI)
Food-Approach Traits		
Enjoyment of food / Food responsiveness	1.51 (1.06, 2.14)*	1.28 (0.79, 2.06)
Emotional overeating	1.56 (1.05, 2.32)*	1.16 (0.67, 2.00)
Desire to drink	1.22 (0.94, 1.60)	1.54 (1.08, 2.20)*
Food-Avoidant Traits		
Slowness in eating / Satiety responsiveness	0.74 (0.52, 1.05)	0.73 (0.45, 1.18)
Food fussiness	0.86 (0.64, 1.15)	0.93 (0.62, 1.39)
Emotional undereating	1.02 (0.73, 1.43)	0.97 (0.62, 1.52)

All models adjusted for ethnicity, household income, maternal age and child BMI z-scores at 5 years old

*Significant at $p < 0.05$

+Reference: Low Food Reward (n = 144)

S4 Table: Associations between food reward at 6 years old and child adiposity at 7 years old (n = 321) (Unimputed)

	Total Skinfolds (mm)	Subscapular (mm)	Triceps (mm)	Biceps (mm)	Suprailiac (mm)	BMI (z-scores)
	β (95% CI)	β (95% CI)	β (95% CI)	β (95% CI)	β (95% CI)	β (95% CI)
High Food Reward+	1.32 (-2.08, 4.72)	0.61 (-0.33, 1.55)	0.34 (-0.64, 1.31)	0.05 (-0.57, 0.67)	0.32 (-0.74, 1.39)	0.14 (-0.16, 0.45)

Adjusted for ethnicity, household income, maternal BMI, maternal age, parity, gestational age

+Reference: Children with Low Food Reward data 7 years old (n = 138)

S5 Table: Associations between food reward at 6 years old and child adiposity at 7 years old (n = 466) (High Food Reward, Low Food Reward, wrote equally)

	Total Skinfolds (mm)	Subscapular (mm)	Triceps (mm)	Biceps (mm)	Suprailiac (mm)	BMI (z-scores)
	β (95% CI)	β (95% CI)	β (95% CI)	β (95% CI)	β (95% CI)	β (95% CI)
High Food Reward+ (n = 228)	1.66 (-1.29, 4.61)	0.60 (-0.21, 1.42)	0.48 (-0.36, 1.31)	0.15 (-0.39, 0.68)	0.43 (-0.50, 1.37)	0.19 (-0.07, 0.45)
Wrote Equally+ (n = 68)	0.03 (-4.17, 4.23)	0.04 (-1.12, 1.19)	0.03 (-1.16, 1.22)	-0.11 (-0.86, 0.65)	0.07 (-1.26, 1.41)	0.03 (-0.35, 0.40)

Adjusted for ethnicity, household income, maternal BMI, maternal age, parity, gestational age

+Reference: Children with Low Food Reward data 7 years old (n = 170)

S6 Table: Associations between food reward at 6 years old and child adiposity at 7 years old (n = 321) (Unimputed)

	Underweight (n = 12)^	Overweight / Obese (n = 80)^
	OR (95% CI)	OR (95% CI)
High Food Reward+	1.69 (0.47, 6.02)	1.32 (0.75, 2.32)

Adjusted for ethnicity, household income, maternal BMI, maternal age, parity, gestational age

+Reference: Children with Low Food Reward data 7 years old (n = 138)

^Reference: Normal weight status at 7 years old (n = 229)

S7 Table: Associations between food reward at 6 years old and child adiposity at 7 years old (n = 466)
(High Food Reward, Low Food Reward, wrote equally)

	Underweight (n = 16)^	Overweight / Obese (n = 106)^
	OR (95% CI)	OR (95% CI)
High Food Reward+ (n = 228)	1.61 (0.46, 5.69)	1.42 (0.85, 2.36)
Wrote Equally+ (n = 68)	3.24 (0.74, 14.23)	0.90 (0.43, 1.91)

Adjusted for ethnicity, household income, maternal BMI, maternal age, parity, gestational age

+Reference: Children with Low Food Reward data 7 years old (n = 170)

^Reference: Normal weight status at 7 years old (n = 344)

S8 Table: Associations between maternal feeding practices at 5 years old and food reward at 6 years old (n = 292) (Unimputed)

	Model 2	*p-interaction*	Male (n = 160)	Female (n = 132)
	OR (95% CI)		OR (95% CI)	OR (95% CI)
Positive Feeding Practices				
Encourage balance/variety	0.95 (0.62, 1.45)	*< 0.01*	0.52 (0.28, 0.96)*	1.80 (0.93, 3.48)
Teaching nutrition	1.35 (0.97, 1.89)	*< 0.01*	0.92 (0.59, 1.43)	2.38 (1.32, 4.28)**
Healthy environment	0.90 (0.64, 1.25)	*0.10*		
Involvement	1.04 (0.80, 1.37)	*0.91*		
Modelling	1.01 (0.79, 1.30)	*0.16*		
Monitoring	0.95 (0.74, 1.21)	*0.47*		
Negative Feeding Practices				
Restriction for health	1.36 (1.02, 1.83)*	*0.71*		
Restriction for weight	1.05 (0.79, 1.39)	*0.32*		
Child control	1.20 (0.80, 1.79)	*0.89*		
Emotion regulation	0.93 (0.67, 1.29)	*0.33*		
Food as a reward	1.06 (0.83, 1.35)	*0.18*		
Pressure to eat	1.13 (0.82, 1.56)	*0.22*		

All models adjusted for ethnicity, household income, maternal age and child BMI z-scores at 5 years old

*Significant at $p < 0.05$; **Significant at $p < 0.01$

S9 Table: Associations between maternal feeding practices at 5 years old and food reward at 6 years old (n = 367) (High Food Reward, Low Food Reward, wrote equally)

	High Food Reward (n = 168)⁺	Wrote Equally (n = 60)⁺	*Wrote Equally p-interaction*	Wrote Equally Male (n = 34)	Wrote Equally Female (n = 26)
	OR (95% CI)	OR (95% CI)		OR (95% CI)	OR (95% CI)
Positive Feeding Practices					
Encourage balance/variety	0.92 (0.61, 1.41)	1.08 (0.61, 1.90)	*0.04*	0.58 (0.26, 1.27)	1.94 (0.78, 4.83)
Teaching nutrition	1.21 (0.88, 1.67)	0.89 (0.58, 1.36)	*0.02*	0.58 (0.33, 1.04)	1.53 (0.75, 3.12)
Healthy environment	0.84 (0.60, 1.16)	0.72 (0.47, 1.12)	*0.54*		
Involvement	1.01 (0.78, 1.32)	1.04 (0.73, 1.49)	*0.65*		
Modelling	0.99 (0.77, 1.26)	0.86 (0.62, 1.20)	*0.40*		
Monitoring	0.97 (0.76, 1.23)	0.94 (0.68, 1.29)	*0.16*		
Negative Feeding Practices					
Restriction for health	1.38 (1.03, 1.85)*	1.51 (1.01, 2.27)*	*0.32*		
Restriction for weight	1.04 (0.79, 1.38)	0.89 (0.61, 1.32)	*0.27*		
Child control	1.25 (0.84, 1.86)	1.63 (0.95, 2.79)	*0.79*		
Emotion regulation	0.93 (0.68, 1.27)	1.15 (0.77, 1.72)	*0.17*		
Food as a reward	1.07 (0.84, 1.36)	1.40 (1.00, 1.96)	*0.95*		
Pressure to eat	1.17 (0.85, 1.61)	1.34 (0.86, 2.06)	*0.04*	0.78 (0.42, 1.43)	2.15 (1.08, 4.27)*

All models adjusted for ethnicity, household income, maternal age and child BMI z-scores at 5 years old

*Significant at $p < 0.05$

⁺Reference: Low Food Reward (n = 139)

S10 Table: Spearman rank correlation of maternal feeding practice at 5 years old among girls (n = 168)

		1	2	3	4	5	6	7	8	9	10	11
1	Child control	-	-	-	-	-	-	-	-	-	-	-
2	Emotion regulation	0.22**	-	-	-	-	-	-	-	-	-	-
3	Encourage balance/variety	-0.16*	-0.05	-	-	-	-	-	-	-	-	-
4	Healthy environment	-0.26**	-0.11	0.35**	-	-	-	-	-	-	-	-
5	Food as a reward	0.10	0.31**	0.04	-0.14	-	-	-	-	-	-	-
6	Involvement	0.01	0.09	0.26**	0.37**	0.17*	-	-	-	-	-	-
7	Modelling	-0.27**	0.07	0.58**	0.48**	0.08	0.34**	-	-	-	-	-
8	Monitoring	-0.20**	-0.12	0.32**	0.21**	-0.01	0.17*	0.28**	-	-	-	-
9	Pressure to eat	-0.06	0.19*	0.28**	0.03	0.19*	0.13	0.25**	-0.13	-	-	-
10	Restriction for health	-0.22**	0.05	0.28**	0.11	0.25**	0.01	0.32**	0.27**	0.18*	-	-
11	Restriction for weight	-0.18*	0.14	0.09	0.16*	0.15*	0.21**	0.22**	0.04	-0.03	0.29**	-
12	Teaching nutrition	-0.12	-0.06	0.59**	0.38**	0.001	0.20**	0.47**	0.32**	0.17*	0.15*	-0.07

**Correlation is significant at the 0.01 level (2-tailed).

*Correlation is significant at the 0.05 level (2-tailed).
